# Nasopharyngeal SARS-CoV-2 viral load kinetics using digital PCR

**DOI:** 10.1016/j.heliyon.2023.e20739

**Published:** 2023-10-06

**Authors:** Elizabeth Hastie, Harold Amogan, David Looney, Sanjay R. Mehta

**Affiliations:** a Division of Infectious Diseases and Global Public Health, University of California, San Diego, 9500 Gilman Drive, CA, 92093, USA; b Veterans Medical Research Foundation, 3350 La Jolla Village Drive, San Diego, CA, 92163, USA; c San Diego Veterans Affairs Medical Center, San Diego, CA, 92163, USA

**Keywords:** Sars-CoV-2, Viral kinetics, Digital PCR, COVID-19, Viral decay

## Abstract

**Background:**

The relationship between the viral kinetics of SARS-CoV-2 and clinical outcomes remains unclear.

**Methods:**

A convenience sample of 955 remnant nasopharyngeal swabs collected during routine care between 11/18/20 and 9/26/21 were analyzed using digital PCR and associated clinical data extracted from the medical record. 18 individuals had >1 sample within 30 days of onset of symptoms.

**Results:**

Paired samples were an average of 6 [range: 0–13] days apart. Four individuals sampled twice on the same day had a median 0.52 log_10_ viral load difference between samples. Of the remaining, 12 individuals had a decrease in viral load over time, with an average decay of −0.23 log_10_/day.

**Conclusions:**

Our study found a similar rate of viral decay to others, but did not find associations between viral kinetics and clinical outcomes. Larger studies would be useful to support the use of this measurement as a surrogate endpoint for therapeutic studies.

## 1 Background

The viral kinetics of SARS-CoV-2 infection in the nasopharynx help provide an understanding of how long individuals remain infectious [1]. While detection of nucleic acid is not equivalent to finding infectious virions, others have shown a relationship between the amount of viral RNA detected and culturable virus [2]. Numerous studies have examined the relationship between viral RNA kinetics and clinical outcomes, with wide variation in the results [3,4]. This may be due to many factors, including but not limited to the SARS-CoV-2 variant, host immune status, vaccination status, and antiviral therapy. In addition, the time from onset of infection, swab location (i.e., anterior nares vs nasopharyngeal (NP)), quality of sampling, and accuracy of the quantification methods also impact the results of kinetic studies. However, such studies are important for understanding the potential impact of antiviral therapy on clinical outcomes.

In RT-qPCR, quantification is achieved by comparing cycle threshold (Ct) values, or the PCR cycle at which fluorescence intensity reaches a specified threshold, to standard curves created from samples with known quantities [5]. This generated result is dependent on standard calibration curves and is not an absolute quantification [[5], [6], [7], [8]]. Digital polymerase chain reaction (dPCR) is a newer quantitative PCR technology that allows for absolute quantification of nucleic acid using Poisson distribution analysis [5,9,10] without the need for a standard curve [7,[10], [11], [12], [13]]. Digital PCR has been shown be more sensitive, specific, and consistent relative to RT-PCR [[6], [7], [8],[10], [11], [12],[14], [15], [16], [17], [18], [19]]. Here, we used dPCR to improve the quality of our viral kinetic study.

### 1.1 Objectives

Here, we present a real-world sample of 18 paired NP swabs where we examined the viral decay kinetics and explored associations with severity of disease, vaccination status and treatment.

### 1.2 Study design

#### 1.2.1 Patients and specimens

A convenience sample of 955 remnant NP swab eluent samples from positive tests collected during routine clinical care at the San Diego Veterans Affairs Medical Center (SDVAMC) between 11/18/20 and 9/26/21. Individuals with more than one positive NP swab collected within 30 days of each other were included in this study for a total of 39 NP swabs: 15 paired NP swabs from individuals with two positive NP swabs and 3 triplet NP swabs from individuals with 3 positive NP swabs (Supplementary Fig. 1). All testing was obtained from veterans receiving care in the Emergency Room, in ancillary outdoor testing facilities, at regional clinics, or in the inpatient setting.

#### 1.2.2 Patient consent statement

This study was approved by the Research Ethics Committee at SDVAMC. The requirement to obtain informed consent was waived by the Ethics Committee.

#### 1.2.3 SARS-CoV-2 RNA testing

NP swabs were collected by trained nurses at testing location and were placed in 3 mL of universal transport medium (UTM). Initial testing for the presence of SARS-CoV-2 was performed in the clinical laboratory using several different platforms including: Cepheid GeneXpert®, Roche Liat®, Roche COBAS® 6800, and BioFire FilmArray®. Remnant samples from positive tests were aliquoted and stored at −80C until further analysis was performed.

#### 1.2.4 Droplet digital PCR

Remnant samples were analyzed using digital PCR (dPCR). Full details of the analysis can be found in Hastie et al. [20] and in the Supplementary Material. In brief, RNA was extracted from 140 μL of the UTM, and eluted from the QIAgen column with 60 μL of Buffer AVE. 1 μL of the RNA extraction was loaded into a reaction mix, droplets were generated using the BioRad QX200 Droplet Generator, and PCR was run overnight using BioRad's C1000 Touch thermal cycler. PCR results were read on the BioRad QX200 Droplet Reader the following day.

#### 1.2.5 Clinical data collection

Chart review was conducted extracting demographic and clinical details. Samples from employees of the SDVAMC were also excluded. Occasionally, subsequent care was obtained outside the SDVAMC. Summarized records of these visits were usually available in the SDVAMC electronic medical record. Only individuals with repeated samples (e.g., repeat testing from the same individual) within 30 days of the onset of symptoms were included in this analysis.

#### 1.2.6 Statistical analysis

Statistical analysis was performed using the R statistical computing program [21]. Associations between continuous variables were examined using parametric (Pearson correlation) and non-parametric (Spearman correlation) approaches. Differences between medians across groups were compared in a pairwise fashion using the non-parametric Mann-Whitney test, given that SARS-CoV-2 RNA loads were non-normally distributed. Other differences between groups were analyzed using the Shapiro. test and t. test functions. Two-sided exact p-values were reported; p < 0.05 was considered statistically significant. Multivariate linear regression performed using the glm function in R. Akaike information criterion (AIC) was used to compare the multivariate models and determine which one was the best fit for the data (i.e., explaining the greatest amount of variation with the fewest number of variables). Graphing was performed using the ggplot2 package [22].

## 2 Results

A total of 18 veterans had multiple positive samples within 30 days of the onset of symptoms for a total of 39 positive nasopharyngeal swabs. Fifteen individuals had two positive swabs and three individuals had 3 positive swabs. Of the 18 veterans, 83.3 % (n = 15) were male. The average age was 55 [range: 28–81]. Sampling occurred at a median of 4.9 [range: 1–14] days after the onset of symptoms, with follow up sampling occurring an average of 6 [range: 0–13] days after the initial sample. Three-quarters of individuals (72.2 %, n = 13) were unvaccinated at diagnosis while 27.8 % (n = 5) were fully vaccinated at the time of diagnosis. Frequency of comorbid conditions are shown in Table 1.

**Table 1:** Descriptive characteristics of cohort.

Characteristic	Description	Number with data available
Age^a^	5.1 ( ± 15.5) years	18
***Gender***		
Male	83.3% (15)	18
***Ethnicity***		
Hispanic/Latino	11.1% (2)	18
***Comorbidity***		
DM2	22.2 % (4)	18
CAD	0% (0)	18
HTN	27.8% (5)	18
Hemodialysis	0% (0)	18
Prior Solid Organ Transplant	0% (0)	18
COPD/Asthma	22.2% (4)	18
BMI^a^	27.9( ± 5.1)	18
***Symptoms***		
Respiratory	94.4% (17)	18
Gastrointestinal	66.6% (12)	18
Fever	66.6% (12)	18
Headache	27.8% (5)	18
Loss of Taste and/or Smell	44.4% (8)	18
***Laboratory Values***		
Peak CRP^b^	12.9(0.38–25.63) mg/dL	7
Peak D-dimer^b^	0.7 (0.5–12.2) mg/L	7
Absolute Lymphocytes (nadir)^b^	0.6 (0.4–1.3) 10^3^/μL	9

BMI= Body mass index, DM2 = Type 2 diabetes mellitus, CAD = coronary artery disease, COPD= Chronic obstructive lung disease, HTN= Hypertension, CRP=C-reactive protein.

a Mean and standard deviation.

b Median and range.

Among the 18 individuals included in the analysis, four had repeat sampling on the same day (one of these individuals had three samples), 12 showed a decrease in the log_10_ nasopharyngeal viral load with time, and three showed a slight increase in the viral load with time. Two of the three individuals that had a slight increase in viral load were sampled at three timepoints. There was not a consistent trajectory when these three viral loads were plotted over time.

To assess reproducibility, we evaluated the viral load differences among samples collected on the same day in the same individuals. Among the pairs of samples meeting this criteria, the median difference in measurements was a 0.52 log _10_ viral load difference [range 0.22–1.51].

**Fig. 1:**
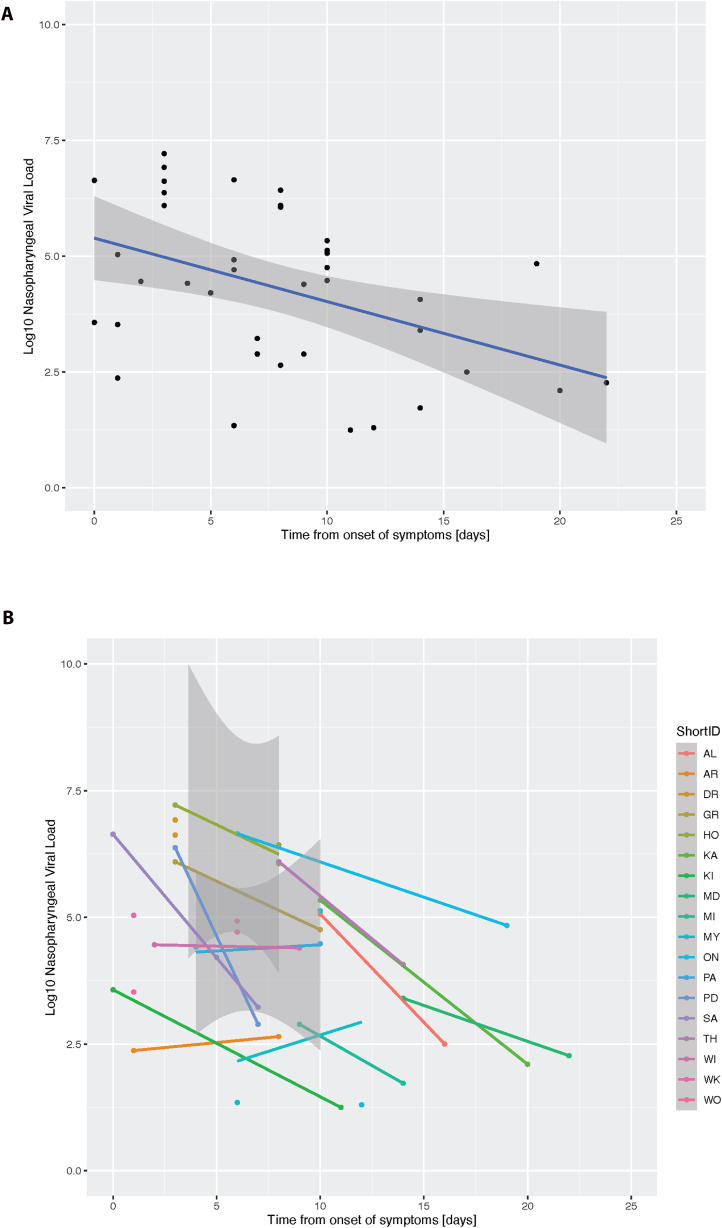
A. Log_10_ Nasopharyngeal SARS-CoV-2 viral load plotted by time from onset of symptoms. 39 samples from 18 individuals were included in the analysis. Equation of the trend line y = −0.14x + 5.4, R_2_ = 0.19. Fig. 1 B. Log_10_ Nasopharyngeal SARS-CoV-2 viral load plotted by time from onset of symptoms with slopes calculated for each of the participants. All 18 individuals are included in this plot including those with repeat samples on the same day.

We next evaluated the overall trajectory of viral load over time including all data points from all 18 individuals on the same graph normalized by the time from the onset of symptoms (Fig. 1A). The rate of decline in the viral load was calculated to be a decline of 0.14 in the log_10_ viral load per day from the onset of symptoms [Standard error ± 0.05, p-value of 0.005, R^2^ = 0.19]. To more accurately evaluate the viral kinetics in this population, we averaged the slope of viral decline (or increase) calculated individually across all individuals included in the cohort (Fig. 1B). The mean change in log_10_ viral load per day was 0.23 [standard deviation ± 0.25].

We next compared the slope of change between different subgroups. We did not observe any differences in the mean slope between persons older vs younger than 50 (−0.20 vs −0.26, P = 0.75). We observed a trend towards slower decline in those with a body mass index (BMI) of greater than 30 compared to less than 30 (−0.1 vs −0.29), which did not reach statistical significance (p = 0.07). The mean change in log_10_ viral load per day was not significantly different between fully vaccinated and unvaccinated individuals (−0.44 vs −0.15, p = 0.15) or those who received antiviral treatment (e.g., remdesivir or monoclonal antibody) vs those who did not (−0.14 vs −0.28, p = 0.27). There was not a significant difference between individuals admitted vs not admitted (−0.16 vs −0.28, p = 0.37). Similarly, we also did not find a significant difference between individuals who required supplemental oxygen vs those who did not (−0.15 vs −0.26, p = 0.44). Shapiro-Wilks testing did not reject the assumption of normality for any of these subgroup comparisons.

## 3 Discussion

Using dPCR technology, we observed a mean nasopharyngeal log_10_ viral load decay rate of 0.23/day (∼1.7-fold daily decline, or not quite 1 cycle threshold) from onset of symptoms. While many studies looking at viral kinetics have used relative quantification methods (e.g. cycle threshold value) without calculating the measured viral load [[23], [24], [25], [26]], our results showed a similar rate of decline compared to the study by Watson et al. [27] in which extensive data collected from by the National Basketball Association occupational health program was examined using anterior nares swabs. This study found the posterior distribution of the estimate for slope in the bi-exponential model to be ∼0.2 log_10_/day.

To further characterize sub-populations within our study cohort, we compared decay rates between persons with risk factors for more severe disease and with adverse outcomes. We did not find significant differences except for a trend toward slower rate of decline in persons with a BMI of >30. The limited sample size of our study cohort limits our ability to make negative conclusions with regards to these factors. Larger studies to evaluate the roles of risk factors in relation to SARS-CoV-2 viral dynamics would be useful to support the use of this measurement as a surrogate endpoint for therapeutic studies.

## Funding

This investigator-initiated project was supported by a generous grant from Gilead Sciences, Inc. This research was also supported by the San Diego Center for AIDS Research (SD CFAR), an NIH-funded program (P30 AI036214), which is supported by the following NIH Institutes and Centers: 10.13039/100000060NIAID, NCI, 10.13039/100000050NHLBI, NIA, NICHD, NIDA, 10.13039/100000072NIDCR, 10.13039/100000062NIDDK, 10.13039/100000057NIGMS, 10.13039/100000025NIMH, 10.13039/100006545NIMHD, FIC, and OAR.

## Institutional Review Board statement

The study was conducted in accordance with the Declaration of Helsinki, and approved by the Institutional Review Board (or Ethics Committee) of San Diego Veterans Affairs Medical Center (#1225657, approval date February 7, 2021).

## Informed consent statement

The requirement to obtain informed consent was waived by the Ethics Committee, as this was a retrospective study utilizing chart review and reanalysis of previously collected samples.

## Data availability statement

Data from this study has not been deposited into a public repository given that it includes protected health information. Data supporting the reported results will be shared upon request by the Dr. Sanjay Mehta (sanjay.mehta2@va.gov).

## CRediT authorship contribution statement

**Elizabeth Hastie:** Formal analysis, Investigation, Writing – original draft, Writing – review & editing. **Harold Amogan:** Investigation, Methodology. **David Looney:** Conceptualization, Formal analysis, Investigation, Writing – review & editing. **Sanjay R. Mehta:** Conceptualization, Data curation, Formal analysis, Funding acquisition, Investigation, Methodology, Project administration, Supervision, Writing – original draft, Writing – review & editing.

## Declaration of competing interest

The authors declare that they have no known competing financial interests or personal relationships that could have appeared to influence the work reported in this paper.

## References

[1] Trunfio M., Richiardi L., Alladio F. (2022). Determinants of SARS-CoV-2 contagiousness in household contacts of symptomatic adult index cases. Front. Microbiol..

[2] Jefferson T., Spencer E.A., Conly J.M. (2022). Viral cultures, cycle threshold values and viral load estimation for assessing SARS-CoV-2 infectiousness in haematopoietic stem cell and solid organ transplant patients: a systematic review. J. Hosp. Infect..

[3] Krifors A., Karlsson L., Ekman M., Lorant C., Skorup P. (2023). The kinetics of SARS-CoV-2 viremia in COVID-19 patients receiving remdesivir. Eur. J. Clin. Microbiol. Infect. Dis..

[4] Neant N., Lingas G., Le Hingrat Q. (2021). Modeling SARS-CoV-2 viral kinetics and association with mortality in hospitalized patients from the French COVID cohort. Proc. Natl. Acad. Sci. U. S. A..

[5] Long S., Berkemeier B. (2022). Ultrasensitive detection and quantification of viral nucleic acids with Raindance droplet digital PCR (ddPCR). Methods.

[6] Kojabad A.A., Farzanehpour M., Galeh H.E.G. (2021). Droplet digital PCR of viral DNA/RNA, current progress, challenges, and future perspectives. J. Med. Virol..

[7] Mio C., Cifu A., Marzinotto S. (2021). Validation of a one-step reverse transcription-droplet digital PCR (RT-ddPCR) approach to detect and quantify SARS-CoV-2 RNA in nasopharyngeal swabs. Dis. Markers.

[8] Sun Y., Ding C., Chen Q. (2021). Digital PCR assay for the effective detection of COVID-19 patients with SARS-CoV-2 low viral load. J. Virol. Methods.

[9] Mathekga B.S.P., Nxumalo Z., Thimiri Govinda Raj D.B. (2022). Micro and nanofluidics for high throughput drug screening. Prog Mol Biol Transl Sci.

[10] Taylor S.C., Laperriere G., Germain H. (2017). Droplet Digital PCR versus qPCR for gene expression analysis with low abundant targets: from variable nonsense to publication quality data. Sci. Rep..

[11] Falzone L., Musso N., Gattuso G. (2020). Sensitivity assessment of droplet digital PCR for SARS-CoV-2 detection. Int. J. Mol. Med..

[12] Nyaruaba R., Mwaliko C., Dobnik D. (2022). Digital PCR applications in the SARS-CoV-2/COVID-19 era: a roadmap for future outbreaks. Clin. Microbiol. Rev..

[13] CfDCa Prevention Test for SARS-CoV-2 only. https://www.cdc.gov/coronavirus/2019-ncov/lab/virus-requests.html.

[14] Li J., Lin W., Du P. (2022). Comparison of reverse-transcription qPCR and droplet digital PCR for the detection of SARS-CoV-2 in clinical specimens of hospitalized patients. Diagn. Microbiol. Infect. Dis..

[15] Liu C., Shi Q., Peng M. (2020). Evaluation of droplet digital PCR for quantification of SARS-CoV-2 Virus in discharged COVID-19 patients. Aging (Albany NY).

[16] Lu R., Wang J., Li M. (2022). Retrospective quantitative detection of SARS-CoV-2 by digital PCR showing high accuracy for low viral load specimens. J Infect Dev Ctries.

[17] Marchio A., Batejat C., Vanhomwegen J. (2021). ddPCR increases detection of SARS-CoV-2 RNA in patients with low viral loads. Arch. Virol..

[18] Milosevic D., Moyer A.M., Majumdar R., Kipp B.R., Yao J.D. (2022). A reverse-transcription droplet digital PCR assay to detect and quantify SARS-CoV-2 RNA in upper respiratory tract specimens. J. Clin. Virol..

[19] Xu J., Kirtek T., Xu Y. (2021). Digital droplet PCR for SARS-CoV-2 resolves borderline cases. Am. J. Clin. Pathol..

[20] Hastie E., Amogan H., Looney D., Mehta S.R. (2023). Association between SARS-CoV-2 viral load and patient symptoms and clinical outcomes using droplet digital PCR. Viruses.

[21] Team R.C.R. (2022). Computing RFfS.

[22] Wickham H. (2016).

[23] Anantharaj A., Gujjar S., Verma N. (2022). Resolution of viral load in mild COVID-19 patients is associated with both innate and adaptive immune responses. J. Clin. Virol..

[24] Kim D.Y., Bae E.K., Seo J.W., Yun N.R., Kim C.M., Kim D.M. (2021). Viral kinetics of severe acute respiratory syndrome coronavirus 2 in patients with coronavirus disease 2019. Microbiol. Spectr..

[25] Long H., Zhao J., Zeng H.L. (2021). Prolonged viral shedding of SARS-CoV-2 and related factors in symptomatic COVID-19 patients: a prospective study. BMC Infect. Dis..

[26] Teyssou E., Marot S., Cocherie T. (2022). Prolonged replication of BA.1 and BA.2 Omicron lineages compared to Delta variant in nasopharyngeal samples from COVID-19 patients. Infect. Dis. Newsl..

[27] Watson J.A., Kissler S.M., Day N.P.J., Grad Y.H., White N.J. (2022). Characterizing SARS-CoV-2 viral clearance kinetics to improve the design of antiviral pharmacometric studies. Antimicrob. Agents Chemother..

