# Supplementary material for: Nasopharyngeal SARS-CoV-2 viral load kinetics using digital PCR
Source: Heliyon. 2023 Oct 6;9(10):e20739. doi: 10.1016/j.heliyon.2023.e20739 (PMC10590800; doi:10.1016/j.heliyon.2023.e20739)
Supplement: Multimedia component 1 [file mmc1.docx]

**Supplementary Material**

*Nasopharyngeal SARS-CoV-2 Viral Load Kinetics Using Droplet Digital PCR*


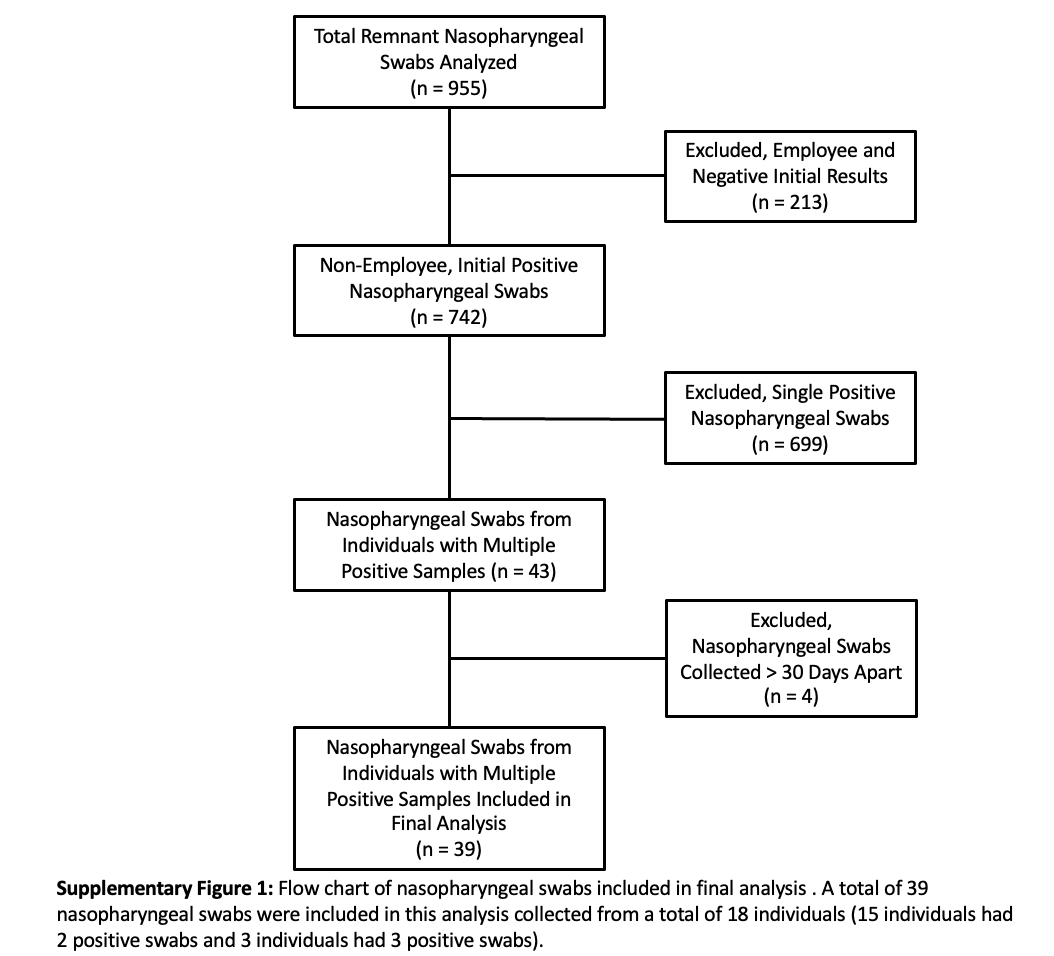


**Supplementary Figure 1:** Flow chart of nasopharyngeal swabs included in final analysis . A total of 39 nasopharyngeal swabs were included in this analysis collected from a total of 18 individuals (15 individuals had 2 positive swabs and 3 individuals had 3 positive swabs).

**Details of Droplet Digital PCR**

RNA was extracted from the samples using the QIAamp Viral RNA Mini kit. Following the kit’s protocol, RNA was extracted from 140µL of sample, and eluted from the QIAgen column with 60µL of Buffer AVE. Nucleic acid concentration was then measured using the NanoDrop instrument.

BioRad’s One-Step RT-ddPCR Advanced Kit for Probes protocol was used to estimate viral load. For ddPCR each sample was prepared in duplicate with the average reported as the viral load. Briefly, 1µL of sample was loaded into a reaction mix, droplets generated from the reaction mix using the BioRad QX200 Droplet Generator, and PCR was run overnight using BioRad’s C1000 Touch thermal cycler. PCR results were then read on the BioRad QX200 Droplet Reader the following day.

Oligonucleotide sequences, PCR settings, and thermal cycler conditions are shown below:

**Primers and probe used in ddPCR:**

**Label Name Oligonucleotide sequence (5’ to 3’)**

| 2019-nCoV_N1-F | GAC CCC AAA ATC AGC GAA AT |
| --- | --- |
| 2019-nCoV_N1-R | TCT GGT TAC TGC CAG TTG AAT CTG |
| 2019-nCoV_N1-P | FAM-ACC CCG CAT/ZEN/TAC GTT TGG TGG ACC-3IABkFQ |

Note: Oligonucleotide sequences were from the Center for Disease Control and Prevention.

**Preparation of PCR mix:**

**Component 1X(µL)**

| Supermix | 5 |
| --- | --- |
| Reverse Transcriptase | 2 |
| 300mM DTT | 1 |
| Forward Primer, 10µM | 1.8 |
| Reverse Primer, 10µM | 1.8 |
| Probe, 2.5µM | 2 |
| Water | 5.4 |
| Sample (80-117ng) | 1 |
| Total | 20 |

**Thermal Cycler Conditions:**

**Temperature, ºC Time Number of Cycles**

| 50 | 60 min | 1 |
| --- | --- | --- |
| 95 | 10 min | 1 |
| 95 | 30 sec (Ramp rate 2 ºC/sec) | 40 |
| 55 | 1 min (Ramp rate 2 ºC/sec) | 40 |
| 98 | 10 min | 1 |
| 4 | Infinite | 1 |

**Assessing Assay Variance**

All samples were in duplicate. We examined variance between runs and within runs.  Within runs, we found that the log10 difference between repeated measures of viral load from the same sample ranged from 0-1.9, with an average 0.08, a SD of 0.18, and a SEM 0.01. The calculated variance (S^2^) value of the difference in Log10 values was 0.0258.  Notably, all 50 samples with more than 100% variation (difference/average) have <250 copies with average of <10 copies. When looking at the variance between runs, we specifically looked at sample 2 for which data was generated on 17 separate runs. The log10 mean of the measured viral load was  4.6543, with a STD 0.227, a SEM 0.0057, and a variance (S^2^) 0.0005.
